# Physicians’ experiences with reporting domestic violence against women: a cross-sectional study in Saudi Arabia

**DOI:** 10.1186/s12245-024-00635-z

**Published:** 2024-04-24

**Authors:** Sarah Almuammar, Wijdan Alhowig

**Affiliations:** Family Medicine Department, Faculty of Medicine, King Abdulaziz University, Jeddah, Saudi Arabia

**Keywords:** Violence, Reporting, Women’s Health, Saudi Arabia

## Abstract

**Background:**

Domestic violence, including violence against women, is a global public health concern with significant implications for women’s health and well-being. Despite its prevalence, healthcare providers often face barriers when reporting cases of domestic violence. This study aims to estimate the prevalence of reporting domestic violence against women by physicians and to explore the barriers to reporting.

**Methods:**

This cross-sectional study was conducted at King Abdulaziz University Hospital in Saudi Arabia. Data were collected through a self-administered questionnaire distributed to physicians from various specialties. The questionnaire covered sociodemographic information, physicians’ experiences with domestic violence cases, and barriers to reporting.

**Results:**

The study included 400 physicians. Approximately 39.8% of physicians reported encountering cases of domestic violence, with 33.0% documenting such cases. Reporting rates varied among occupational levels, with specialists (50.85%) and consultants (38.78%) reporting more frequently than general practitioners (16.67%) and residents (28.93%). Physicians with over 10 years of experience were more likely to report (49.40%, *p* = 0.001). Among the various categories of barriers examined, a lower score in physician-related barriers was the only category significantly associated with higher reporting rates (*p* < 0.01). However, health administration- and victim-related barriers were not significant factors in reporting.

**Conclusion:**

This study highlights variations in reporting rates among medical specialties and underscores the positive impact of physician experience on reporting domestic violence cases. Addressing physician-related barriers and promoting a reporting culture are crucial steps toward combating domestic violence in Saudi Arabia. Healthcare providers play a vital role in identifying and addressing this public health issue.

## Background

Domestic violence, a pressing and preventable public health concern, encompasses a range of violent behaviors, including physical, sexual, psychological aggression, and stalking, often involving family members and controlling conduct [1, 2]. Violence against women, a subset of domestic violence, is defined by the United Nations as “any act of gender-based violence causing physical, sexual, or psychological harm or suffering to women, including threats of such acts, coercion, or arbitrary deprivation of liberty” [3]. It is a widespread issue affecting nearly one in three women worldwide, with approximately 30% experiencing physical or sexual intimate partner violence or sexual violence by non-partners during their lifetime [4]. Intimate partner violence, in particular, poses significant threats to women’s health and well-being in both the short and long term [5].

The prevalence of intimate partner violence varies across regions, with reported rates ranging from 22% in high-income countries and Europe to 33% in the Americas and the Eastern Mediterranean region [4]. In Saudi Arabia, recent studies have highlighted the prevalence of domestic violence, particularly in the Western Region, including Makkah, Jeddah, and Al-Taif, where it was reported to be as high as 33.24% [2]. A study in 2017 involving 758 Saudi women from 13 governorates revealed an intimate partner violence prevalence of 32% [6]. To address this issue, the Saudi Government introduced the Law for Protection from Abuse in 2013, aimed at providing comprehensive protection, assistance, shelter, and support for survivors of abuse. The Ministry of Social Affairs established the Domestic Violence Protection Program and a national reporting hotline for abuse cases.

Healthcare providers, including physicians, play a critical role in addressing domestic violence, as they often encounter victims or potential victims while providing care to women and children [7]. Identifying domestic violence early and offering appropriate interventions can mitigate the severe physical and psychological consequences of abuse [7]. However, despite their essential role, the reporting of domestic violence cases by physicians remains relatively low. Several factors contribute to this reluctance, including inadequate training, time constraints, lack of awareness, women’s hesitancy to disclose abuse, and the perception of domestic violence as a social issue beyond the healthcare providers’ purview [7, 8].

Against this backdrop, this study seeks to explore the barriers faced by physicians when it comes to reporting domestic violence against women. Understanding these barriers is crucial for developing effective strategies to enhance reporting and support for survivors.

## Methods

### Study design

This cross-sectional observational study aimed to investigate the barriers that physicians encounter when reporting cases of domestic violence against women at King Abdulaziz University Hospital, a major tertiary care hospital in the Western region of Saudi Arabia.

### Study settings

King Abdulaziz University Hospital is one of the largest hospitals in Saudi Arabia, with a total bed capacity of over 850. It serves as a prominent healthcare institution staffed with approximately 4,000 healthcare providers, including physicians, nurses, and various healthcare professionals. Located in Jeddah, Saudi Arabia, King Abdulaziz University Hospital plays a pivotal role in delivering advanced medical care and education, making it an ideal setting for examining physicians’ barriers to reporting domestic violence against women.

### Study participants

To ensure a representative sample, we employed a stratified random sampling technique among physicians at King Abdulaziz University Hospital. Within each medical specialty, a proportionate sample of physicians was randomly selected using a simple random sampling technique, guaranteeing diversity within the sample. The minimum required sample size, calculated to achieve a desired level of precision with a 95% confidence level, was set at 300 physicians. This calculation was based on data from hospital employment records.

### Data collection

Physicians at all levels, both male and female, who had experience dealing with domestic violence, were invited to participate in this study. Data collection was conducted through a self-administered questionnaire distributed via an online survey link. The questionnaire was designed to be easily accessible and convenient for participants, preserving their anonymity throughout the data collection process. This online survey link was disseminated to physicians through various channels, including social media applications and email communications.

### Survey instrument

The survey questionnaire consisted of four sections:

#### Section 1: sociodemographic characteristics

This section collected essential sociodemographic information, including sex, age, nationality, educational qualification, occupation level, medical specialty, marital status, and years of professional experience.

#### Section 2: physicians dealing with domestic violence cases

This section assessed participants’ perceptions of domestic violence against women and inquired whether they had previously documented such cases in patients’ health records.

#### Section 3: barriers to reporting domestic violence

This section identified three primary domains of barriers:

1. **Physician-Related Barriers**: These included insufficient training, fear of offending patients, fear of abusers, limited knowledge of domestic violence signs, beliefs about social acceptance, perceiving it as a private matter, past reporting experiences, feelings of hopelessness, and encounters with patients’ relatives during examinations.
2. **Health Administration-Related Barriers**: These encompassed the absence of training programs, legal knowledge gaps, time constraints, heavy physician workloads, limited encouragement from health authorities, and the absence of clear reporting policies, including the definition of domestic violence.
3. **Victim-Related Barriers**: These involved concerns about child custody, fears of negative judgments from healthcare professionals and the community, cultural and religious barriers like shame, embarrassment, guilt, ignorance about abuse, and doubts about physicians’ effectiveness in addressing domestic violence.

The survey questionnaire used in the study assessed barriers to reporting domestic violence against women among physicians. Participants were asked to rate their agreement or disagreement with various statements representing different types of barriers related to physicians, health administration, and victims. Each barrier category included multiple items, and participants provided responses on a Likert scale (e.g., agree, uncertain, disagree). For each barrier category, a barrier score was calculated by summing the responses to the corresponding items. A lower barrier score indicated fewer perceived barriers to reporting, while a higher score indicated more perceived barriers.

#### Section 4: optional open-ended questions

The final section provided participants with the option to share additional insights on barriers that might impede physicians from reporting cases of domestic violence against women.

### Statistical analysis

Descriptive statistics were employed to summarize the data, presenting means ± standard deviations for continuous variables and frequencies with percentages for categorical variables. Categorical data were compared using the chi-squared test, while continuous data were assessed using t-tests. The internal consistency of the barriers was evaluated using Cronbach’s alpha test, and the item scores for each barrier were summed for further analysis. Additionally, three sets of binary logistic regression analyses, adjusted for age, sex, and years of experience, were conducted to examine the impact of each barrier on previous reporting of domestic violence. Reporting odds ratios (ORs) and their respective 95% confidence intervals (CIs) were calculated. To prevent multicollinearity, the regression model excluded occupational level. All statistical analyses were performed using Stata software version 15.1, with statistical significance set at an alpha level of 0.05.

### Ethical considerations

Ethical approval for this study was obtained from the Unit of Biomedical Ethics at King Abdulaziz University (Reference No 586 − 21). Informed consent was secured from all participants, ensuring their understanding of the study’s purpose, voluntary participation, and the right to withdraw at any point.

## Results

### Sociodemographic and work characteristics

This study included 400 physicians, and Table 1 summarizes their sociodemographic and work-related characteristics. The mean age of participants was 30.41 ± 6.33 years, with 56.75% being female. Approximately half of the physicians were single, and only 1.75% were divorced. The majority of participants, 94.25%, were Saudi nationals. Occupational levels consisted of 3.00% general practitioners, 70.00% residents, 14.74% specialists, and 12.25% consultants. Concerning years of experience, 69% had less than 5 years, while around 20% had 10 or more years of experience.

**Table 1:** Profile of physicians: sociodemographic and work-related characteristics

Characteristics		*N*	(%)
Age (mean ± SD)		30.41 ± 6.33	
Gender	Male	173	43.25
	Female	227	56.75
Nationality	Saudi	377	94.25
	Non-Saudi	23	5.75
Occupational Level	General Practitioner	12	3.00
	Resident	280	70.00
	Specialist	59	14.74
	Consultant	49	12.25
Speciality	Emergency Medicine	23	5.75
	General Practice	124	31.00
	Internal Medicine	98	24.50
	Obstetrics and Gynaecology	11	2.75
	Paediatrics	26	6.50
	Psychiatry	17	4.25
	Radiology	10	2.50
	Surgery	86	21.50
	Other	5	1.25
Years of Experience	< 5	276	69.00
	5–10	41	10.25
	≥10	83	20.75

Abbreviations: *N*: number of participants

### Reporting of domestic violence

Overall, 159 (39.8%) physicians reported that they had encountered cases of domestic violence during their practice. Notably, 132 (33.0%) physicians acknowledged documenting cases of domestic violence against women in patient health records.

Barriers to reporting domestic violence were assessed within three domains: physician-related, health administration-related, and victim-related. The mean score for physician-related barriers, which could potentially range from 10 to 30, was 19.88 ± 4.12. Health administration-related barriers, which had a potential range from 6 to 18, had a mean score of 15.18 ± 2.21. Victim-related barriers, which had a potential range from 2 to 6, had a mean score of 4.85 ± 1.25.

### Factors associated with reporting domestic violence

Table 2 illustrates the associations between sociodemographic and work-related characteristics and domestic violence reporting. While no significant differences were observed in age or gender, physicians who reported domestic violence tended to be older. Notably, there were significant disparities in reporting based on occupational levels, with specialists (50.85%) and consultants (38.78%) reporting domestic violence more frequently compared to general practitioners (16.67%) and residents (28.93%). Variations also existed across medical specialties, with psychiatrists (76.47%) and obstetricians/gynecologists (63.64%) having higher reporting rates. Additionally, 49.40% of physicians who reported domestic violence had over 10 years of experience (*P* < 0.01).

**Table 2:** Relationships between sociodemographic and work-related characteristics

Characteristics		Had Previous Reporting of Domestic Violence				*P* value
		No (*N* = 268)		Yes (*N* = 132)		
		*n* (%)		*n* (%)		
Age (mean ± SD)		29.77 ± 5.99		31.70 ± 6.81		0.99
Gender	Male	116	(67.05)	57	(32.95)	0.98
	Female	152	(66.96)	75	(33.04)	
Nationality	Saudi	254	(67.37)	123	(32.63)	0.52
	Non-Saudi	14	(60.87)	9	(39.13)	
Occupational Level	General practitioner	10	(83.33)	2	(16.67)	**0.01**
	Resident	199	(71.07)	81	(28.93)	
	Specialist	29	(49.15)	30	(50.85)	
	Consultant	30	(61.22)	19	(38.78)	
Speciality	Emergency Medicine	13	(56.52)	10	(43.48)	**< 0.01**
	General Practice	96	(77.42)	28	(22.58)	
	Internal Medicine	61	(62.24)	37	(37.76)	
	Obstetrics and Gynaecology	4	(36.36)	7	(63.64)	
	Paediatrics	20	(76.92)	6	(23.08)	
	Psychiatry	4	(23.53)	13	(76.47)	
	Radiology	8	(80.00)	2	(20.00)	
	Surgery	59	(68.00)	27	(31.40)	
	Other	3	(60.00)	2	(40.00)	
Years of Experience	> 5	200	(72.46)	76	(27.54)	**< 0.01**
	5–10	26	(63.41)	15	(36.59)	
	≥10	42	(50.60)	41	(49.40)	
Barriers Score (mean ± SD)	Physician-Related	20.31 ± 3.85		19.00 ± 4.52		**< 0.01**
	Health Administration-Related	15.25 ± 2.25		15.03 ± 2.12		0.17
	Victim-Related	4.93 ± 1.26		4.68 ± 1.21		**0.02**

Abbreviations: *N*: number of participants, *SD*: standard deviation

Regarding different barriers, significant differences in mean scores between reporters and non-reporters emerged for both physician- and victim-related barriers. Interestingly, the mean scores for both types of barriers were lower among the reporters (*P* < 0.01 and *P* = 0.02, respectively).

### Determinants of reporting domestic violence: multivariable regression analysis models

Table 3 presents the results of three regression analyses evaluating the association between each barrier and previous reporting of domestic violence. All models were adjusted for age, sex, and years of experience. Among the three barriers factors, only physician-related barriers were significantly associated with previous reporting of domestic violence (OR = 0.92, 95% CI: 0.87–0.97). Furthermore, increasing age (OR = 1.04, 95% CI: 1.01–1.08) and over 10 years of experience (OR = 2.56, 95% CI: 1.55–4.25) were determinants of previous reporting of domestic violence. However, in the other models, neither health administration- nor victim-related barriers were significantly associated with previous reporting of domestic violence.

**Table 3:** Adjusted regression analyses: barriers and previous reporting of domestic violence

Model	Variable		Multivariable Regression Model		
			OR	*P* value	95% CI
Physician-Related Barriers	Age		1.04	**0.01**	1.01–1.08
	Gender	Male	0.99	0.98	0.65–1.51
		Female	Reference Group		
	Years of Experience	> 5	Reference Group		
		5–10	1.51	0.23	0.76–3.02
		≥10	2.56	**< 0.01**	1.55–4.25
	Physician-Related Barriers		0.92	**< 0.01**	0.87–0.97
Health Administration-Related Barriers	Age		1.05	**0.04**	1.01–1.11
	Gender	Male	0.87	0.56	0.56–1.36
		Female	Reference Group		
	Years of Experience	> 5	Reference Group		
		5–10	0.61	0.40	0.19–1.93
		≥10	1.95	**0.02**	1.09–3.49
	Health Administration-Related Barriers		0.92	0.31	0.86–1.04
Victim-Related Barriers	Age		1.05	**0.03**	1.01–1.11
	Gender	Male	0.88	0.57	0.56–1.36
		Female	Reference Group		
	Years of Experience	> 5	Reference Group		
		5–10	0.63	0.43	0.20–1.97
		≥10	1.90	**0.02**	1.06–3.41
	Victim-Related Barriers		0.85	**0.06**	0.71–1.00

Abbreviations: *OR*: odds ratio; *CI*: confidence interval

## Discussion

Domestic violence against women is a global public health concern that has garnered significant attention from both medical and social perspectives [1]. In the Kingdom of Saudi Arabia, domestic violence against women is a pressing issue, affecting nearly one-third of women and being associated with factors such as lower education levels and substance abuse by spouses [9]. While domestic violence against women is increasingly recognized as a critical problem, there has been a dearth of research investigating the barriers that physicians face when reporting such incidents. Our study sought to address this gap by exploring these barriers from the perspective of physicians working in a university hospital in Western Saudi Arabia.

One noteworthy finding from our study was the variation in reporting rates among physicians of different specializations. Specialists and consultants reported domestic violence more frequently compared to general practitioners and residents. This discrepancy suggests that specialists and consultants may be more oriented towards the implications of domestic violence, potentially due to higher levels of training and knowledge. To bridge this gap, implementing targeted training programs for physicians with lower reporting rates is essential. These programs can enhance awareness regarding domestic violence and encourage reporting, ultimately contributing to the improvement of women’s health and the well-being of the community. Additionally, the highest number of reports of domestic violence came from psychiatrists, followed by obstetricians and gynecologists. The nature of their work likely contributes to this pattern, as psychiatrists regularly address psychological issues, and the connection between domestic violence and psychological distress is well-established [10]. Furthermore, the preponderance of female obstetricians and gynecologists in Saudi Arabia, driven by cultural and religious factors [11], may make them more adept at recognizing signs of domestic violence than their male counterparts. This aligns with findings from Reibling et al. [12], which indicated that female physicians reported more domestic violence cases than males.

Another significant finding was that more experienced physicians were more likely to report domestic violence incidents than those with less experience. This observation is consistent with expectations, as experienced physicians may have received more training in dealing with domestic violence, possess greater knowledge of the topic, and have encountered more domestic violence cases in their careers. Similar patterns were observed by Reibling et al. [12], who found that older and more experienced physicians were more inclined to report domestic violence cases.

The significance of our findings lies in elucidating the factors associated with previous reporting of domestic violence among healthcare providers. Our analysis revealed that among the three barrier factors investigated—physician-related, health administration-related, and victim-related—only physician-related barriers were significantly associated with previous reporting. This indicates the pivotal role of physician-related factors, such as training adequacy and concerns about offending patients, in influencing reporting behaviors. Understanding these differences in perception is crucial for tailoring interventions and support strategies effectively. By addressing specific barriers identified among non-reporter can foster an environment conducive to reporting and ultimately improve the identification and support of domestic violence survivors.

Physician-related barriers to reporting domestic violence were more common among pediatricians and family physicians compared to emergency physicians. Family physicians, in particular, often miss opportunities to screen women for domestic violence across various clinical situations [13]. While pediatricians are uniquely positioned to evaluate and screen for domestic violence, this practice is seldom implemented [14]. In contrast, emergency physicians are frequently confronted with severe domestic violence cases [15], leading to an expected higher reporting frequency in this group. Additionally, fewer health administration-related barriers were observed among emergency medicine physicians and psychiatrists compared to physicians from other specialties. The discrepancy in health administration-related barriers observed among emergency medicine physicians and psychiatrists compared to physicians from other specialties may be attributed to several factors. Emergency medicine physicians often work in fast-paced, high-pressure environments where prompt decision-making and intervention are crucial, potentially leading to a greater emphasis on addressing urgent health concerns such as domestic violence. Additionally, emergency departments frequently implement standardized protocols and procedures for identifying and responding to cases of domestic violence, which may reduce administrative barriers associated with reporting. Similarly, psychiatrists, who specialize in mental health and behavioral disorders, may receive specialized training in recognizing and addressing psychosocial issues such as domestic violence, leading to a greater awareness and comfort level in reporting such cases.

Several limitations should be considered when interpreting our findings. First, our study was conducted at a single healthcare facility, which may limit the generalizability of our results to other settings. Second, the absence of similar studies for direct comparison necessitated an interpretation of our findings based on our own insights. Third, the cross-sectional design of our study precludes establishing causality between independent and dependent variables, as it only demonstrates associations. Lastly, the utilization of self-administered questionnaires in our research may introduce response bias, potentially leading to over- or underestimations of reported barriers. Despite these limitations, our study holds public health significance by shedding light on the barriers to reporting domestic violence and their determinants, aiming to address these barriers and promote the reporting of this critical issue in our community.

## Conclusion

In conclusion, this study provides valuable insights into the barriers faced by physicians when reporting domestic violence against women in a university hospital in Western Saudi Arabia. Our findings reveal variations in reporting rates among different medical specialties, emphasizing the need for targeted training programs to enhance awareness and reporting among physicians with lower reporting rates. Additionally, the positive correlation between reporting and physicians’ experience underscores the importance of continued education and training in this area. Addressing physician- and victim-related barriers and promoting a culture of reporting are essential steps toward combating domestic violence against women in Saudi Arabia. These findings contribute to the broader discourse on domestic violence prevention and intervention, highlighting the critical role of healthcare providers in identifying and addressing this public health issue.

## Data Availability

The datasets generated and/or analyzed during the current study are available from the corresponding author upon reasonable request.
